# Non-Equilibrium Casimir Force between Vibrating Plates

**DOI:** 10.1371/journal.pone.0053228

**Published:** 2013-01-10

**Authors:** Andreas Hanke

**Affiliations:** Department of Physics, University of Texas at Brownsville, Brownsville, Texas, United States of America; German Cancer Research Center, Germany

## Abstract

We study the fluctuation-induced, time-dependent force between two plates confining a correlated fluid which is driven out of equilibrium mechanically by harmonic vibrations of one of the plates. For a purely relaxational dynamics of the fluid we calculate the fluctuation-induced force generated by the vibrating plate on the plate at rest. The time-dependence of this force is characterized by a positive lag time with respect to the driving. We obtain two distinctive contributions to the force, one generated by diffusion of stress in the fluid and another related to resonant dissipation in the cavity. The relation to the dynamic Casimir effect of the electromagnetic field and possible experiments to measure the time-dependent Casimir force are discussed.

## Introduction

A fundamental advance in the understanding of nature was the insight that physical forces between bodies, instead of operating at a distance, are generated by *fields*; the latter obeying their own dynamics, implying a finite speed of propagation of signals and causality [1]. Moreover, time-varying fields can sustain themselves in otherwise empty space to produce disembodied waves; exemplified by electromagnetic fields and waves, and gravitational fields. Gravitational waves are believed to be detected in the near future [2].

Another force seemingly operating at a distance is the Casimir force. This force was first predicted by Casimir in 1948 for two parallel conducting plates in vacuum, separated by a distance 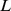, for which he found an attractive force per unit area 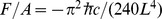 [3]. It can be understood as resulting from the modification of quantum-mechanical zero-point fluctuations of the electromagnetic field due to confining boundaries [4]–[7]. In the last decade, high-precision measurements of the Casimir force have become available which confirm Casimir's prediction within a few per cent [8]–[11]; recent experiments demonstrate the possibility of using the Casimir force as an actuation force for movable elements in nanomechanical systems [10]–[12]. The thermal Casimir force, generated by thermal rather than quantum fluctuations of the electromagnetic field, has recently been confirmed [13]. This development goes along with significant advances in calculating the Casimir force for complex geometries and materials [7], [14]–[21]. A force analogous to the electromagnetic Casimir force occurs if the fluctuations of the confined medium are of thermal instead of quantum origin [5], [22], [23]. The thermal analog of the Casimir effect, referred to as critical Casimir effect, was first predicted by Fisher and de Gennes for the concentration fluctuations of a binary liquid mixture close to its critical demixing point confined by boundaries [24]; recently, the critical Casimir effect was quantitatively confirmed for this very system [25]. (For computational methods concerning the calculation of critical Casimir forces, see, e.g., Refs. [26]–[28].)

The vast majority of work done on the Casimir effect, and fluctuation-induced forces in general, pertain to the equilibrium case. That is, the system is in its quantal ground state in case of the electromagnetic Casimir effect, or in thermodynamic equilibrium in case of the thermal analog. A number of recent experiments probe the Casimir force between moving components in nanomechanical systems [10]–[12], and effects generated by moving boundaries have been studied, e.g., for Casimir force driven ratchets [29]; however, the data are usually compared with predictions for the Casimir force obtained for systems at rest, corresponding to a quasi-static approximation.

Distinct new effects occur if the fluctuating medium is driven out of equilibrium. In this case the observed effects become sensitive to the dynamics governing the fluctuating medium, which may lead to a better understanding of these systems and may provide new control parameters to manipulate them [30]–[40]. The generalization of the electromagnetic Casimir effect to systems with moving boundaries, referred to as dynamic Casimir effect, exhibits friction of moving mirrors in vacuum and the creation of photons [41]–[44]. Related effects due to oscillating media [45], and nonequilibrium Casimir-Polder forces on moving atoms [46], have also been considered. Interesting effects occur if each body immersed in the fluctuating electromagnetic field is at a different temperature [47], [48]. The associated nonequilibrium Casimir forces and heat transfer between the bodies lead to observable effects [49], [50]. For the thermal analog, fluctuation-induced forces in non-equilibrium systems have been studied in the context of the Soret effect, which occurs in the presence of an external temperature gradient [31]. Effects of temperature changes in classical free scalar field theories and thermal drag forces have been studied in [32]–[35]; however, recently it was argued that the method presented in [32]–[35] is invalid to obtain the fluctuation-induced force exerted on an inclusion or plate embedded in the medium, whereas the stress tensor method, as used in the present work, yields the correct force [40]. Fluctuation-induced forces have also been obtained for macroscopic bodies immersed in mechanically driven systems [30], granular fluids [36], and reaction-diffusion systems [37]. Recently it was shown that non-equilibrium fluctuations can induce self-forces on single, asymmetric objects, and may lead to a violation of the action-reaction principle between two objects [38].

**Figure 1:**
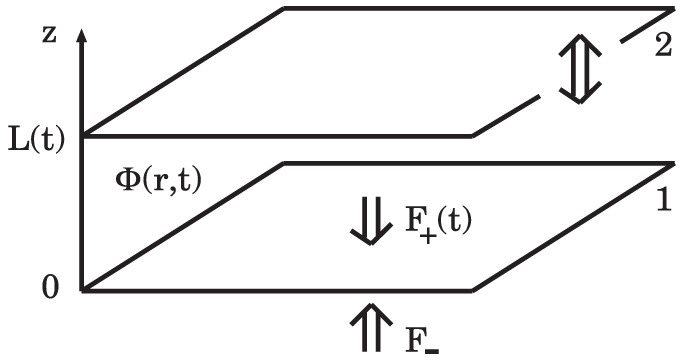
Geometry of two parallel plates separated by a varying distance 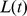
 **.** Plate 1 is at rest while plate 2 is vibrating parallel to the 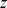-direction. The plates are immersed in a fluctuating medium with purely relaxational dynamics described by a non-conserved scalar order parameter 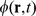. The fluctuation-induced, time-dependent net force 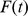 on plate 1 is the sum of forces 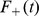 and 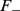 acting on opposite sides of the plate.

In this work we consider a correlated fluid driven out of equilibrium mechanically by a vibrating plate, and study the resulting fluctuation-induced, time-dependent force 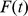 on a second plate at rest. We wish to study the time-dependence of this force in view of the finite speed of diffusion of perturbations in the fluctuating medium, and causality. We consider the simplest possible dynamics of the medium between the plates, namely the purely relaxational dynamics of model A. Specifically, we consider two infinitely extended plates parallel to the 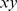-plane, where plate 1 is at rest while plate 2 is vibrating parallel to the 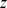-direction by some external driving, resulting in a time-dependent separation (see Fig. 1)
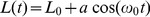
(1)with amplitude 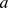 and driving frequency 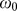. The fluctuating medium is described by a non-conserved scalar order parameter field 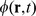, corresponding to the critical dynamics of model A [51], [52], subject to Dirichlet boundary conditions 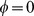 at the plates. The field 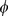 may describe the order parameter of a fluid thermodynamically close to a critical point, or a massless Goldstone mode arising from the breaking of a continuous symmetry such as in nematic liquid crystals or superfluid ^4^He [5], [22]. Our results hold in Gaussian approximation right at the critical point 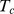 for which the bulk correlation length 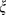 diverges. For 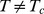 the fluctuation-induced force also depends on the finite correlation length 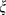. Moreover, for near-critical fluids and binary liquid mixtures the boundary conditions at confining walls correspond to the so-called normal rather than Dirichlet surface universality class [22]. However, it should be noted that dynamic critical behavior is less universal than equilibrium critical behavior. For example, for the liquid-gas transition and the demixing transition in a binary liquid mixture a conserved order parameter convects with the conserved transverse momentum current of the fluid, resulting in critical dynamics referred to as model H [53]; conversely, the superfluid transition of ^4^He corresponds to critical dynamics of model F [54]. In addition, critical dynamics of real fluids may be modified by effects due to gravity and the coupling of the order parameter to secondary densities, which further complicates a quantitative comparison of theory and experiment (see [51], [52] for reviews). Strictly speaking, purely relaxational dynamics of a non-conserved scalar order parameter corresponding to model A only applies to uniaxial magnetic systems and simple lattice gases. However, the results and conclusions derived here for model A dynamics yield new insight in non-equilibrium behavior and may serve as a starting point for more realistic models.

Our results for the time-dependent force 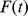 on plate 1 hold to first order in 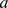 (cf. Eq. (1)). As shown in Fig. 1, 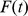 is the sum of forces 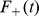 and 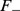 acting on opposite sides of the plate; 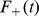 being the force acting on plate 1 from the side of the cavity, and 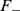 the (time-independent) force on the boundary surface of a semi-infinite half-space filled with the fluctuating medium. The net force 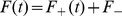 is expected to be finite and overall attractive, i.e., directed towards plate 2.

## Results

### Relaxational Dynamics

In traditional studies of the fluctuation-induced force between two plates, both plates are assumed to be at rest at constant separation 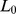 (see Fig. 1). The system is in thermal equilibrium and the fluctuations of the order parameter 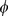 are described by the statistical Boltzmann weight 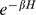 with Gaussian Hamiltonian
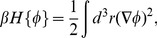
(2)where 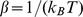 with the Boltzmann constant 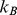 and the temperature 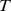 (assumed to be constant). The fluctuation-induced force 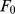 on plate 1 per unit area 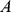 is found to be [5], [22], [23]

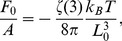
(3)where the minus sign indicates that the force is attractive. Equation (3) is a universal result, independent of the underlying dynamics of the fluctuating medium, as long as the equilibrium is described by Eq. (2).

We now turn to the case where plate 2 is vibrating parallel to the 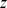-direction, resulting in a time-dependent separation 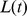 between the plates (see Eq. (1) and Fig. 1). The time-dependent boundary conditions for the order parameter 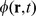 in the medium between the plates now drive the system out of equilibrium. Locally, the order parameter will relax back to equilibrium according to the dynamics of the medium; in this work, we consider a purely relaxational dynamics described by the Langevin equation (see, e.g., Chapter 8 in Ref. [55], and references cited therein)
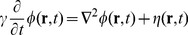
(4)where 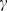 is the friction coefficient. The random force 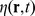 is assumed to have zero mean and to obey the fluctuation-dissipation relation
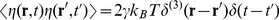
(5)where the brackets 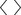 denote a local, stochastic average and 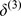 is the delta function in 3 dimensions.

### Non-Equilibrium Casimir Force

The force per unit area acting on plate 1 from the side of the cavity can be expressed as 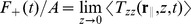 where 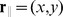 are the components of 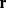 parallel to the plate and 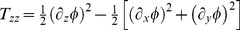 is the 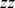-component of the stress tensor [6], [56] (see text below Eq. (104) in Ref. [56] for a discussion of the stress tensor in connection with critical dynamics). Similarly, the force per unit area acting on the other side of plate 1 is given by 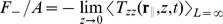 where 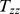 is again evaluated in the cavity between the plates but for the limit 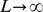 (see Fig. 1). The net force per unit area on plate 1 yields as
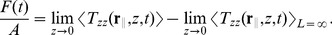
(6)Using the Dirichlet boundary condition 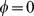 at the plates we obtain
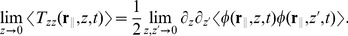
(7)To calculate the two-point correlation function of 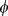 on the right-hand side of Eq. (7) we note that the solution 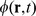 of Eq. (4) can be expressed as
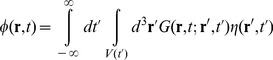
(8)where 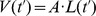 is the volume of the cavity at time 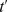 and the Green's function 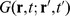 is defined as the solution of
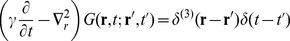
(9)subject to the boundary condition 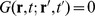 whenever 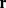 or 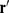 is located on the surface of one of the plates (note that 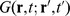 is symmetric in 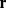 and 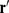). In addition, 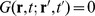 for 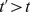 by causality. Thus, 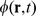 can be expressed as a linear superposition of contributions from the source 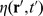 at times 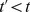 and positions 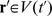, carried forward in time by the propagator 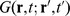. Using Eqs. (8) and (5) one finds the two-point correlation function
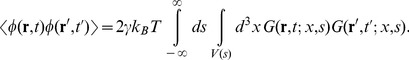
(10)In the present set-up, the system is translationally invariant in 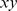-direction at any time 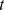, whereas translation invariance in time is broken due to the varying separation 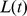 between the plates. Thus, introducing the partial Fourier transform 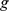 of 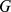 as
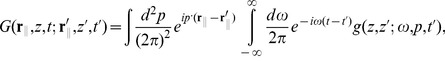
(11)the function 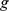 depends explicitly on one of the time coordinates in 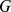, say, 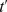. Using Eqs. (10), (11) we find for the expression in Eq. (7) (the star symbol indicates the complex conjugate for real-valued argument 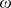)
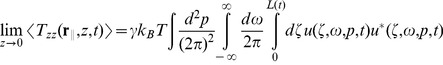
(12)where
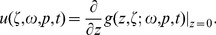
(13)For given propagator 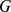, hence function 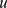, 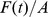 is obtained using Eqs. (6) and (12) (cf. Methods).

### Diffusion of Stress and Resonant Dissipation

The ratio 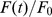 of the fluctuation-induced, time-dependent force 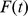 on plate 1 due to the vibrating plate 2 and the corresponding static force 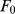 is a universal (cutoff-independent) function of 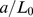 (geometry), 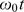 (time-dependence of the driving), and the dimensionless parameter
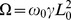
(14)(see Eqs. (1), (3), (4), and Fig. 1). Our results for 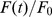 correspond to an expansion to first order in 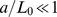 and can be cast in the form
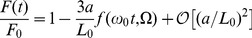
(15a)where the dimensionless function 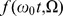 is normalized such that 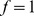 for 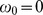. For 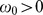 the function 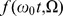 can be represented as
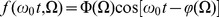
(15b)in terms of an amplitude 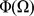 and a phase shift 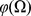.

**Figure 2:**
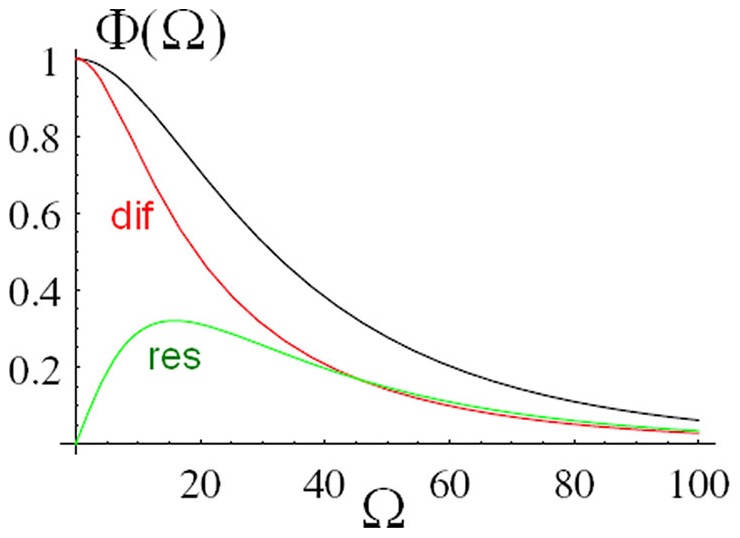
Amplitude of the non-equilibrium Casimir force. Amplitude 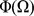 of 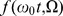 as a function of 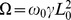 (see Eq. (15)) (black line). Also shown are the amplitudes 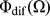 of 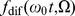 (dif, red line) and 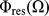 of 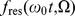 (res, green line) describing contributions to 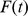 due to diffusion of stress and resonant dissipation, respectively (see [Disp-formula pone.0053228.e140] Eqs. (17), [Disp-formula pone.0053228.e142] (18)).

The amplitude 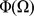 is shown in Fig. 2. For 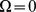, i.e., 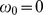, the normalization 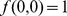 in conjunction with 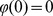 (see below) implies 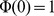. For 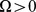, i.e., 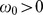, the length 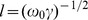 is a measure of the distance over which a perturbation diffuses during an oscillation. For increasing 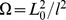 a variation of stress generated at the vibrating plate 2 is more and more attenuated, i.e., washed out, due to the diffusive nature of the medium before it reaches plate 1; thus 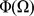 is monotonically decreasing for increasing 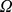 (Fig. 2, black line).

The force 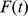 in [Disp-formula pone.0053228.e104] Eq. (15) has contributions of different physical origin related to diffusion of stress (dif) and resonant dissipation (res) in the medium between the plates, i.e.,
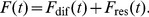
(16)The contributions 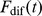 and 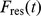 are related to real and imaginary poles in the complex-frequency plane occurring in the calculation of 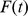, respectively (see Eqs. (35) and (37)). Both 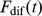 and 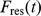 may be expanded as in [Disp-formula pone.0053228.e104] Eq. (15):
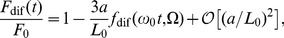
(17a)

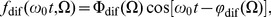
(17b)and
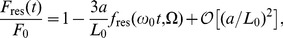
(18a)

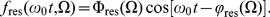
(18b)The amplitude 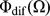 is shown as the red line in Fig. 2. For 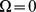, i.e., 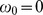, there is no contribution from resonant dissipation, thus 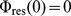 and 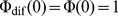 (however, 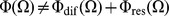 for 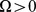 because 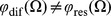). For 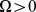 the amplitude 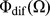 is attenuated due to the diffusive nature of the medium as discussed in relation to 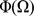 above; thus 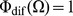 is monotonically decreasing for increasing 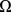 (Fig. 2, red line). Finally, the amplitude 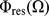 due to resonant dissipation is shown as the green line in Fig. 2. Resonant dissipation is absent in the static case 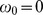, i.e., 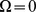, which implies 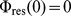. For large 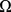 the amplitude 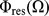 is attenuated due to the diffusive nature of the medium as discussed in relation to 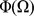 above; thus 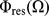 starts at 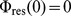, is increasing for small 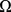 and monotonically decreasing for large 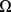 (Fig. 2, green line).

**Figure 3:**
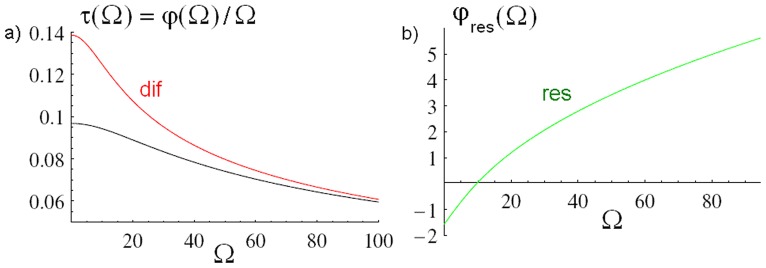
Phase shift of the non-equilibrium Casimir force. (a) Phase shift 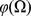 of 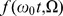 in terms of 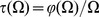 (see [Disp-formula pone.0053228.e104] Eq. (15)) (black line). Also shown is the phase shift 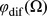 of 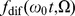 describing the contribution to 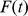 due to diffusion of stress (dif, red line) (see [Disp-formula pone.0053228.e140] Eq. (17)). (b) Phase shift 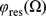 of 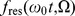 describing the contribution to 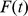 due to resonant dissipation (res) (see [Disp-formula pone.0053228.e142] Eq. (18)).

Figure 3 shows the phase shift 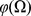 of the function 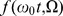 in Eq. (15b) in terms of the variable 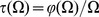. Thus, using Eq. (14), 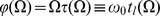, where the lag time 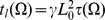 is a measure of the time a variation of stress generated at the vibrating plate 2 takes to diffuse through the medium to reach plate 1. For illustration, for 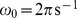 and 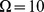 we obtain 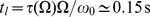. 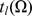 is fairly constant over a wide range of 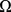 and approaches a finite value 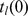 for 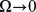 (Fig. 3a, black line); thus 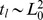 as expected for the present diffusive dynamics in the medium between the plates. In the limit 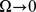 the relation 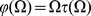 with finite 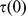 implies 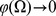. A qualitatively similar behavior occurs for the phase shift 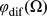 of the function 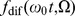 in Eq. (17b) (Fig. 3a, red line). Finally, Fig. 3b shows the phase shift 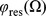 of the function 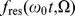 in Eq. (18b); in this case, 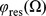 itself is approximately constant and approaches a finite value for 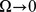 (in contrast to 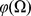, for which 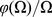 is approximately constant, see above). This implies that the corresponding lag time 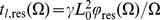 formally diverges for 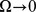, i.e., 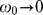, which reflects the fact that resonant dissipation is absent in the static case 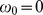. However, the divergence of 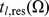 for 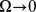 is suppressed in the net force 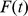 since the amplitude 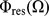 in Eq. (18b) vanishes for 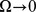, so that the lag time 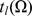 of 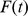 stays finite for 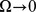 (see [Disp-formula pone.0053228.e104] Eq. (15) and Fig. 3a).

## Discussion

We have studied the fluctuation-induced, time-dependent force 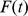 between two plates confining a fluid which is driven out of equilibrium mechanically by harmonic oscillations of one of the plates, assuming purely relaxational dynamics of the fluid (corresponding to the critical dynamics of model A [51], [52]) (see Fig. 1). Our main results for 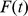, valid to first order in the amplitude 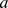 of the oscillations, are summarized in Figs. 2 and 3. We find two distinct contributions to 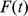 related to diffusion of stress in the fluid and resonant dissipation, respectively. Resonant dissipation has been studied for the dynamic Casimir effect of the electromagnetic field, where it is a result of enhanced creation of photons if the driving frequency corresponds to a resonance frequency of the cavity [41]–[44]. In the present case, dissipation is generated by the viscosity of the fluid described by the friction parameter 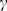 in the Langevin equation (4).

Fluctuation-induced forces may be observed, e.g., by means of atomic force microscopy (AFM). To avoid the experimental difficulty of keeping two flat plates parallel one usually employs geometries in which one of the surfaces is curved; for example, by measuring the force between a sphere attached to the tip of an AFM cantilever and a flat plate. The force 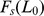 on a sphere of radius 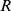 separated by a distance 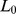 surface-to-surface from a flat plate is related to the energy of interaction per surface area 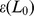 between two flat plates by the proximity force rule 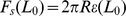. For example, the static force per unit area 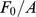 in Eq. (3) yields 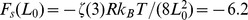 PT for 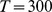 K, 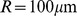, 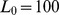 nm, which is readily accessible by AFM.

Fluctuation-induced forces between moving objects are expected to occur for any medium exhibiting long-ranged correlations. However, as mentioned in the Introduction, for real fluids the model needs to be modified to take into account conservation of the order parameter and its convection with the transverse momentum current of the fluid, thus treating Casimir and hydrodynamic interactions on the same footing (corresponding to the critical dynamics of model H [53]); these effects are important and will modify the result for 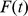 in [Disp-formula pone.0053228.e104] Eq. (15) obtained for the purely relaxational dynamics of model A (see [51], [52] for reviews on dynamic critical behavior and its comparison with experiments). However, the time-dependent force 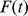 predicted in [Disp-formula pone.0053228.e104] Eq. (15) may be observable by means of computer simulations of the ferromagnetic Ising model (or corresponding lattice gas models) confined between two plates, one of which is vibrating at small amplitude.

A much-studied subject related to the present study are hydrodynamic interactions of microscopic objects in viscous fluids since they are relevant, e.g., to the motility and locomotion of swimming microorganisms [57]. Recently, motivated by devices such as the AFM, the drag experienced by a cylindrical object (modeling an AFM cantilever) and a sphere oscillating at small amplitude near a flat surface were studied in detail [58], [59]; however, few results are available concerning the hydrodynamic force generated by an oscillating object on a *different* object nearby. Thus, it would be interesting to probe effective hydrodynamic interactions between different, moving objects immersed in a viscous fluid, and how these interactions are modified by the Casimir force when correlations in the fluid become long-ranged.

The fluctuation-induced, time-dependent force 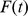 on plate 1 due to the vibrating plate 2 is universal in the sense that it is largely independent of microscopic details of the system. The force ratio 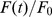, where 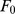 is the force at fixed separation 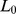, only depends on the dimensionless variables 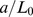 (geometry), 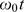 (time-dependence of the driving), and 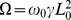 characterizing the viscosity of the fluid. It would be interesting to generalize our approach to the electromagnetic field to study the analogous, time-dependent Casimir force 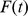 for the dynamic Casimir effect of the electromagnetic field.

## Methods

### The Propagator 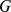


The calculation of the non-equilibrium Casimir force 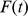 by Eqs. (6) and (12) requires the propagator 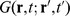 solving Eq. (9) subject to the time-dependent boundary conditions due to the vibrating plate 2. This problem can be solved, for general modulations of the plate(s) in space and time, by the method developed in Refs. [60], [61]. For the present set-up, we find for the partial Fourier transform 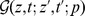 of 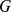 (i.e., transforming the spatial coordinates 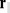, 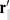 parallel to the plates as in Eq. (11) but keeping the time coordinates 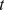, 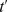; in what follows, we omit the argument 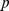 for ease of notation) [60], [61]

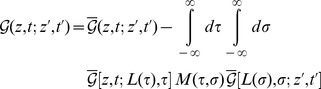
(19)where 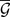 is the propagator in the half-space 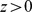 bounded by a Dirichlet surface at 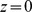 (that is, the function 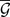 itself is independent of the vibrating plate 2; the dependence of 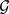 on plate 2 in Eq. (19) only enters through the arguments 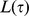, 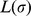 in 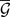, and the kernel 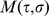). The kernel 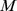 is defined by
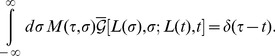
(20)


In this work, we consider small variations of the separation between the plates about a mean separation 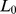, i.e.,
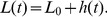
(21)Our results hold to first order in 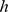. To this end, we insert Eq. (21) in Eq. (19) and expand everything to first order in 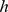 (note that 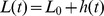 also enters the upper boundary of the integration over 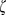 in Eq. (12)). This results in expansions 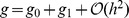 and 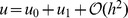 of the functions 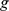 and 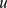 from Eqs. (11), (13) in powers of 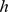. Equations (6), (12) then yield the corresponding contributions to 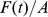.

**Figure 4:**
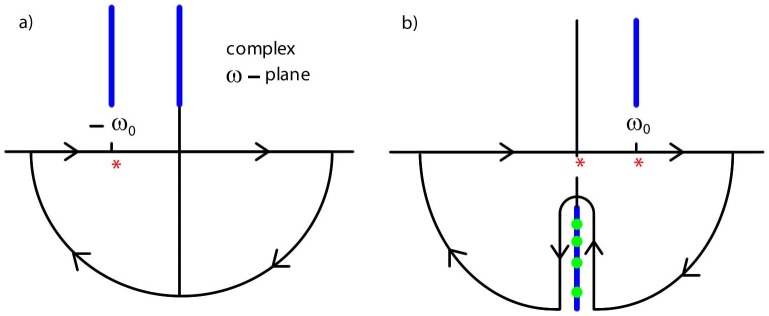
Contour integration in the complex 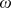
 **-plane.** (a) Contour integration for the second term in square brackets in Eq. (33). The only contribution from this term is from the pole at 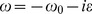 indicated by the red star. The blue lines indicate branch cuts of 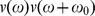. (b) Contour integration in Eq. (36). The blue lines indicate branch cuts of of 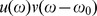. The contributions from the poles at 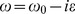 and 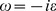 cancel (see text).

Let us first consider the leading order, i.e., 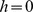 and 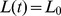. Using Eq. (19) and transforming to 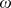-space as in Eq. (11) we find (omitting the arguments 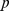 and 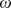 for ease of notation)
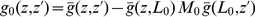
(22)where 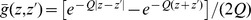 with 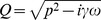 from Eq. (26) below and 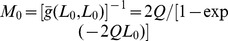. Thus,
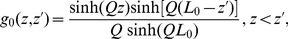
(23)and, using Eq. (13),
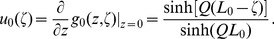
(24)Using Eqs. (6), (12), (24) we thus obtain to leading order [31]

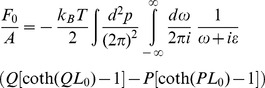
(25a)

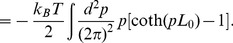
(25b)The integral in Eq. (25b) is finite and yields Eq. (3). In Eq. (25a) we use
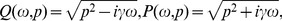
(26)so that 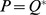 if 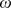 is real. Integrations over 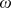 as in Eq. (25a) are readily computed by contour integration in the complex 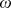-plane. In Eq. (25a) and throughout this work we use the convention that in 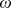-integrations we integrate *above* the pole in 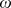; this can be accomplished by the replacement 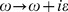 in the denominator of the integrand in Eq. (25a). The limit 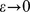 in final results is always understood. Note that this prescription introduces a positive time direction and ensures causality. 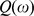 has a branch cut along the negative imaginary axis 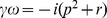, 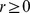, whereas 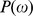 has a branch cut along the positive imaginary axis 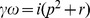, 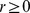. The integral over 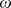 in Eq. (25a) has two contributions. For the contribution involving 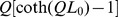, the contour integral can be closed in the upper complex 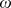-plane (thus avoiding the branch cut of 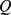), where this term has no poles, so that the contribution from this term vanishes. Likewise, for the contribution involving 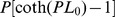, the contour integral can be closed in the lower complex 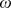-plane (avoiding the branch cut of 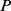), where, in turn, this term has no poles. The single pole at 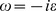 in the lower complex 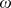-plane then yields the expression in Eq. (25b); cp. Fig. 4a with 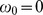.

We now turn to the contribution to 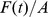 to first order in 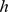. Using the expansion
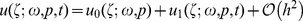
(27)in Eq. (12), with 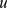 from Eq. (13) and 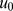 from Eq. (24), we find for general 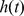


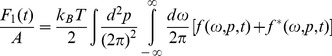
(28)where
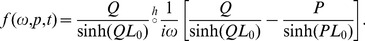
(29)The symbol 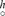 denotes a convolution of two functions 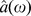, 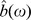 involving an insertion of 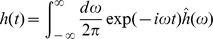:
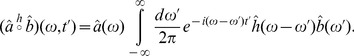
(30)For functions 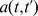, 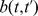, the expression 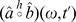 is the representation in 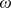-space of 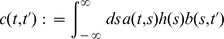; i.e., 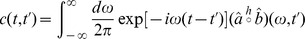. The functions 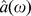, 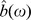 are the representations in 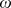-space of 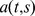, 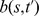, respectively. Equations (28), (29) are obtained by using Eq. (19) with 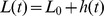, expanding everything to first order in 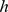, and using Eq. (30) for the resulting insertions of 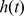. The contribution of 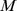 to first order in 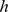 is determined by Eq. (20), resulting in 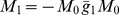 where the subscripts 0 and 1 indicate the order in 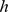; 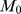 is given below Eq. (22).

For the special case that plate 2 is vibrating with harmonic oscillations of amplitude 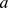 and frequency 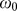 (see Eqs. (1), (21)), i.e.,
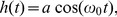
(31)we obtain 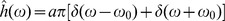. The integral 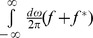 in Eq. (28) decays into two contributions corresponding to the terms in square brackets on the right-hand side of Eq. (29):
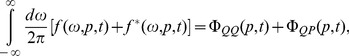
(32)where the subscripts QQ and QP indicate the contributions from the first and second term in square brackets of Eq. (29), respectively. In what follows we show that these two terms yield distinct contributions to 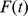 corresponding to real-valued and imaginary poles in the complex 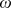-plane.

### Real-Valued Frequency Poles: Diffusion of Stress and Finite Lag Time

For the first contribution in Eq. (32) we find (the 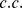 symbol indicates the complex conjugate of the preceding expression; regarding the replacement 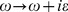 in the denominator of the integrand, see the discussion below Eq. (26))
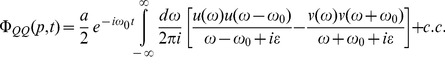
(33)where
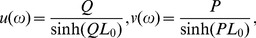
(34)with 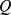, 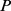 from Eq. (26). Computing the right-hand side of Eq. (33) by contour integration in the complex 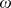-plane, the contributions from the two terms in square brackets in the integrand are analyzed along similar lines as discussed below Eq. (26). Thus, for the first term in square brackets, the contour integral can be closed in the upper complex 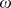-plane, where 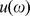 has no poles, so that the contribution from this term vanishes. For the second term in square brackets, the contour integral can be closed in the lower complex 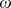-plane, where 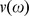 has no poles. The only contribution from this term is from the single pole at 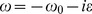; see Fig. 4a. Thus, including the contribution from the complex conjugate in Eq. (33), we obtain
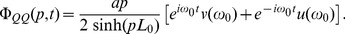
(35)The corresponding contribution to 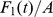 is given by 
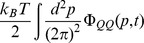
 (see Eqs. (28) and (32)). In the static case, where 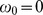 and 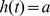 in Eq. (1), this result can also be obtained directly from Eq. (25b) by replacing 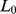 with 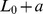 and expanding to first order in 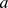. For finite 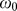, Eq. (35) emerges from the static case by a shift from 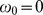 to a finite value of 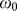. This shift may be understood in terms of a transition from stationary modes in the cavity in the static case to modes with a time-dependence 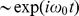 in response to the vibrating plate 2. The resulting fluctuation-induced force 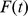 on plate 1 is characterized by a finite lag time 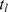 which is a measure of the time a variation of stress in the medium generated at the vibrating plate 2 takes to diffuse through the medium to reach plate 1 (see Fig. 1). For the present diffusive dynamics, 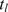 is related to the distance 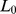 between the plates by 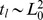.

### Imaginary Frequency Poles: Resonant Dissipation

For the second contribution in Eq. (32) we find
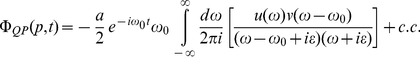
(36)The contour integral over 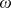 can be closed either in the upper or the lower complex 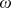-plane, yielding identical results; the contributions from the poles at 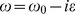 and 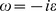 in the lower complex 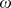-plane cancel. Closing the contour integral in the lower plane, the integral picks up contributions from the imaginary poles 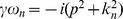 of 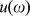, where 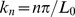 and 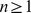 is a positive integer. Note that 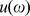 has a branch cut along the negative imaginary axis on which the poles 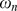 are located (see the related discussion below Eq. (26)); however, this branch cut may be cured using the identity 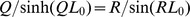, with 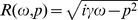, which holds close to the negative imaginary axis. The expression 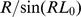 is analytic in the lower complex 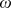-plane with isolated poles at 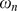; see Fig. 4b. Summing over the residues of these poles yields
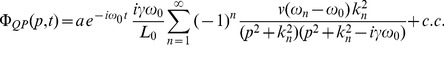
(37)The corresponding contribution to 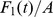 is given by 
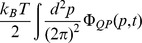
. Note that 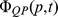 is proportional to 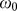, which implies that this term is absent in the static case 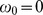 and solely generated by the fact that the system is driven out of equilibrium by the vibrating plate 2 (see Fig. 1). The imaginary-frequency poles 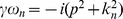 leading to Eq. (37) are related to resonant dissipation in the cavity, where the spectrum of imaginary resonance frequencies 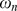 is continuous due to the presence of the continuous in-plane wave number 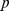 (compare the related discussion of resonant dissipation in the context of the dynamic Casimir effect of the electromagnetic field in Ref. [43]). Resonant dissipation has been studied for the dynamic Casimir effect of the electromagnetic field, where it is a result of enhanced creation of photons if the driving frequency corresponds to a resonance frequency of the cavity [41]–[44].
